# Vascular Ehlers–Danlos syndrome with cryptorchidism, recurrent pneumothorax, and pulmonary capillary hemangiomatosis-like foci: A case report

**DOI:** 10.1097/MD.0000000000008853

**Published:** 2017-11-27

**Authors:** Min A. Park, So Youn Shin, Young Jin Kim, Myung Jae Park, Seung Hyeun Lee

**Affiliations:** a Division of Pulmonary and Critical Care Medicine, Department of Internal Medicine, Kyung Hee University School of Medicine; b Department of Radiology, Kyung Hee University Hospital, College of Medicine, Kyung Hee University; c Department of Laboratory Medicine, Kyung Hee University School of Medicine, Seoul, Republic of Korea.

**Keywords:** case report, *COL3A1* gene, cryptorchidism, Ehlers–Danlos syndrome, ground glass opacities pulmonary capillary hemangiomatosis-like foci, pneumothorax

## Abstract

**Rationale:**

Vascular Ehlers–Danlos syndrome (vEDS) is a rare autosomal dominant inherited collagen disorder caused by defects or deficiency of pro-alpha 1 chain of type III procollagen encoded by *COL3A1*. vEDS is characterized not only by soft tissue manifestations including hyperextensibility of skin and joint hypermobility but also by early mortality due to rupture of arteries or vital organs. Although pulmonary complications are not common, vEDS cases complicated by pneumothorax, hemothorax, or intrapulmonary hematoma have been reported. When a patient initially presents only with pulmonary complications, it is not easy for clinicians to suspect vEDS.

**Patient concerns:**

We report a case of an 18-year-old high school student, with a past history of cryptorchidism, presenting with recurrent pneumothorax.

**Diagnoses:**

Routine laboratory findings were unremarkable. Chest high resolution computed tomographic scan showed age-unmatched hyperinflation of both lungs, atypical cystic changes and multifocal ground glass opacities scattered in both lower lobes. His slender body shape, hyperflexible joints, and hyperextensible skin provided clue to suspicion of a possible connective tissue disorder.

**Interventions:**

The histological examination of the lung lesions showed excessive capillary proliferation in the pulmonary interstitium and pleura allowing the diagnosis of pulmonary capillary hemangiomatosis (PCH)-like foci. Genetic study revealed *COL3A1* gene splicing site mutation confirming his diagnosis as vEDS.

**Outcomes:**

Although his diagnosis vEDS is notorious for fatal vascular complication, there was no evidence of such complication at presentation. Fortunately, he has been followed up for 10 months without pulmonary or vascular complications.

**Lessons:**

To the best of our knowledge, both cryptorchidism and PCH-like foci have never been reported yet as complications of vEDS, suggesting our case might be a new variant of this condition. This case emphasizes the importance of comprehensive physical examination and history-taking, and the clinical suspicion of a possible connective tissue disorder when we encounter cases with atypical presentation and/or unique chest radiologic findings especially in young patients.

## 1 Introduction

Ehlers–Danlos syndrome (EDS) is a group of disorders that affect the connective tissues that support the skin, bones, blood vessels, and many other organs and tissues. EDS has diverse clinical manifestations such as hyperextensibility of skin, hypermobility of joints, tissue fragility, and easy bruising.^[1]^ EDS is classified into 6 types according to clinical and genetic differences; classic, hypermobility, vascular, kyphoscoliotic, arthrochalasia, and dermatosparaxis.^[2]^ Vascular Ehlers–Danlos syndrome (vEDS, also known as type IV EDS) is characterized by thin transparent skin, moderate hyperflexibility of small joints, and fragility of blood vessels and vital organs. The rupture of large artery and bowel in vEDS is associated with early mortality.^[3]^ Although not usually related to issues of mortality, pulmonary complications including pneumothorax, hemothorax, and intrapulmonary hematoma have been documented, predominantly in young adults diagnosed with vEDS.^[4–13]^ However, there is no literature reporting pulmonary capillary hemangiomatosis (PCH)-like foci occurred in patient with vEDS. This case report was approved by Institutional Review Board of Kyung Hee University Hospital.

## 2 Presenting concerns

An 18-year-old male visited our clinic complaining of chest pain and dyspnea of 2 days’ duration.

## 3 Clinical findings

He has a documented history of 2 incidents of spontaneous pneumothorax; a right-sided collapse 8 months prior to the current visit, and a left-sided collapse 3 months ago, which were treated with tube thoracostomy. He had received septoplasty due to nasal septal deviation, and orchiopexy due to right side cryptorchidism 1 year before. He was a high school student who reported that he never smoked and denied any history of significant familial disease.

## 4 Diagnostic focus and assessment

**Figure 1:**
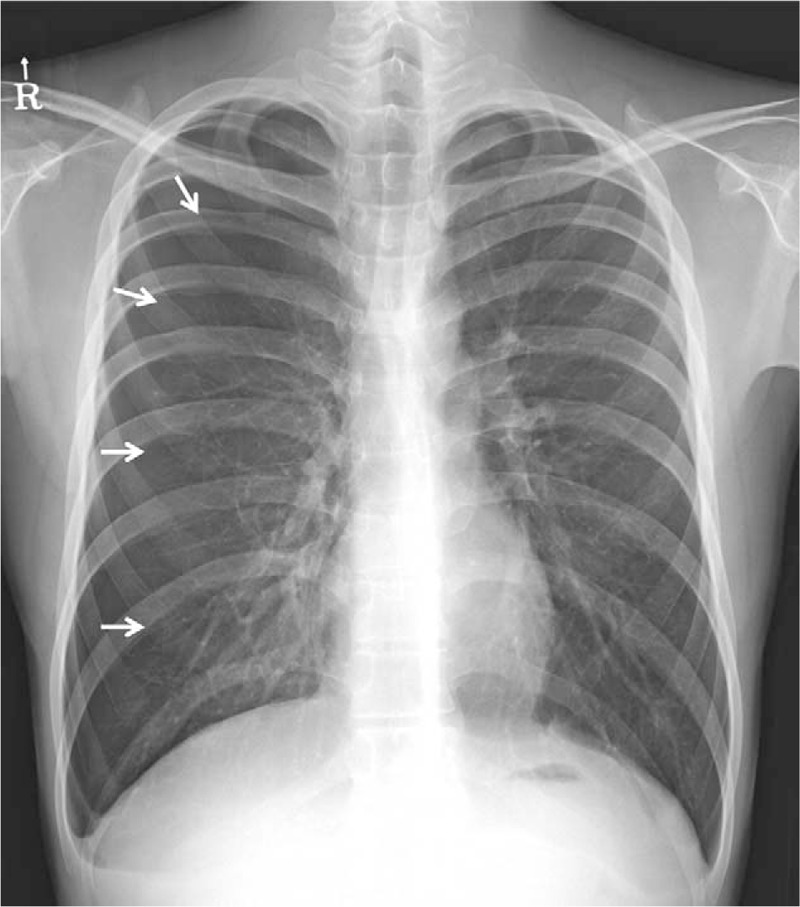
Chest X-ray at presentation. Pneumothorax, which accounts for about 40% of the right hemithorax, was observed. Arrows indicate pleural line.

**Figure 2:**
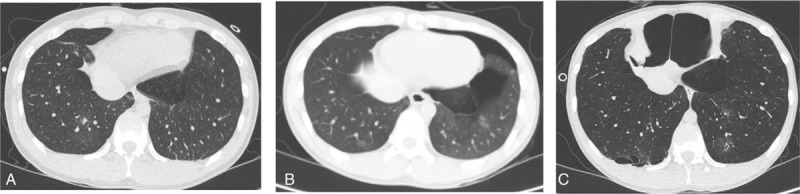
Serial chest CT scans. (A) HRCT scan taken 7 months before this admission (1st attack of pneumothorax-right side) shows nonspecific GGOs in both lower lobes. (B) Conventional CT scan taken 3 months before this admission (2nd attack of pneumothorax-left side) shows the GGOs with waxing and waning pattern compared with previous HRCT. (C) HRCT scan taken after bullectomy for the treatment of 3rd attack of pneumothorax (right side) shows more prominent GGOs and new cystic lesions (arrows) mainly in lower lobes and periphery. Hyperinflation with low attenuation in both lung parenchyma and the resultant interval change in the shape of thorax were also noted. CT = computed tomographic, GGO = ground glass opacity, HRCT = high resolution computed tomography.

His vital signs were stable and oxygen saturation was 95% in ambient air. Chest auscultation revealed decreased lung sound on right hemithorax with normal heart sound. Laboratory test showed white blood cell count of 6920/μL (neutrophil 58.6%, lymphocyte 23.2%, and eosinophil 8.3%), hemoglobin of 14.3 g/dL, and platelet count of 319,000/μL. The serum creatinine, liver function test, and the C-reactive protein level were normal. Test for human immunodeficiency virus was negative. Chest X-ray showed right-sided pneumothorax (Fig. 1) and chest tube was inserted. At hospital day 3, we performed bullectomy using video-assisted thoracoscopic surgery for his recurrent pneumothorax. The surgical specimen was compatible with emphysematous bullae. Despite the surgery, however, air leakage continued and thus pleurodesis was done using 50% dextrose water. The air leakage did not decrease over the ensuing 2 weeks, not even after additional pleurodesis. Chest high resolution computed tomography (HRCT) scan was taken and it showed hyperinflated both lungs with abnormally low attenuation in the lung parenchyma, multifocal ground glass opacities (GGOs), and new cystic lesions predominantly in lower lobes and peripheral portion (Fig. 2C). Compared to the previous computed tomographic (CT) scan taken 3 and 7 months earlier (Fig. 2A, B), the waxing and waning GGOs were becoming more pronounced and the cystic lesions were newly developed. Those findings led us to suspect a variety of rare pulmonary diseases including eosinophilic pneumonia, Langerhans cell histiocytosis, vasculitis or other interstitial lung diseases.

## 5 Follow-up and outcomes

Based on the findings of chest HRCT, further diagnostic investigations were performed. Laboratory tests for auto-antibodies including antinuclear, antineutrophil cytoplasmic, anticardiolipin, antiglomerular basement membrane, and anticitrullinated peptide antibodies resulted negative. Levels of complements were also normal. Bronchoscopy was then performed to check possible endobronchial lesion and to obtain bronchoalveolar lavage fluid (BALF). There was no visible endobronchial lesion. BALF analysis revealed red blood cell count of 2800/μL and white blood cell count of 293/μL (neutrophil 12%, lymphocyte 9%, eosinophil 1%, and macrophage 78%), which was suspicious of occult intrapulmonary hemorrhage. No organisms including parasites or malignancies were detected by culture and cytology.

**Figure 3:**
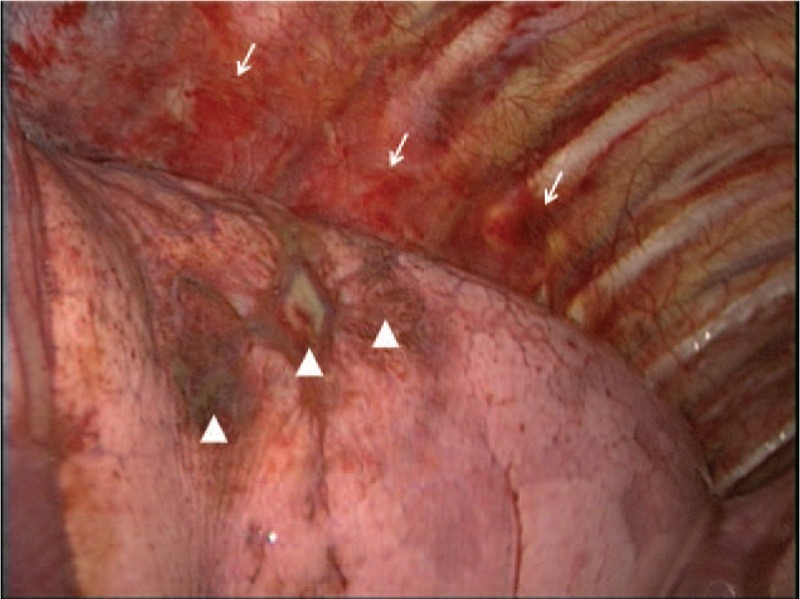
Gross findings during video-assisted thoracoscopic lung biopsy. Hypervascular and hemorrhage-like change on the parietal pleura and multifocal bruise-like lesions on the surface of lung parenchyma were noted (arrows and arrowheads, respectively).

**Figure 4:**
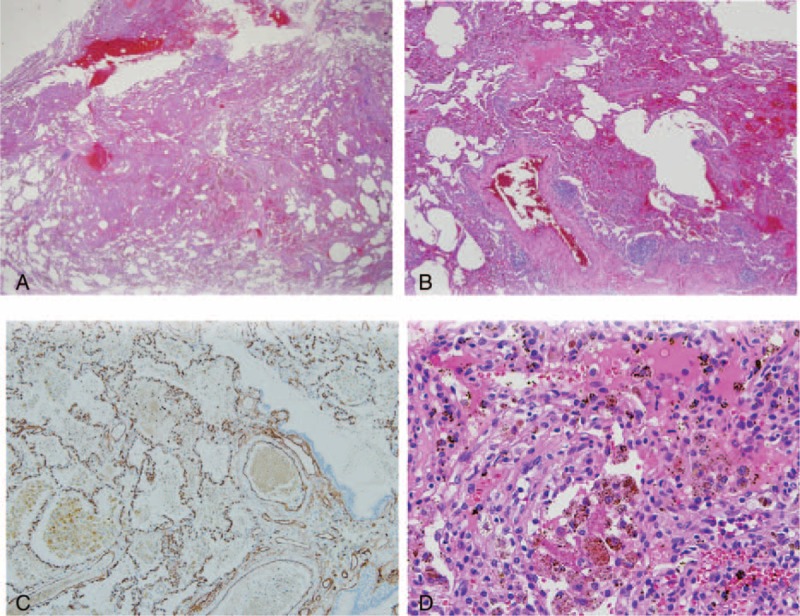
Histopathologic findings of the resected lung tissue. (A, B) Low power view shows excessive capillary proliferation and dilated vessels with some hemorrhage (×50 and ×100, respectively). (C) Immunohistochemical staining for CD34, an endothelial cell marker, supports the proliferation of capillary (×200). (D) The excessive interstitial capillary hyperplasia and hemosiderin-containing macrophages were observed in high power view, which is compatible with pulmonary capillary hemangiomatosis (PCH)-like foci (×400).

To identify the atypical lung lesions in both lower lobes, we performed 2nd video-assisted thoracoscopic surgery lung biopsy. During the operation, the surgeon observed hemorrhage-like changes of the parietal pleura, as well as several bruise-like lesions on the surface of lung parenchyma (Fig. 3). Histopathology revealed interstitial and pleural capillary proliferation with pleural fibrosis and intraalverolar hemosiderin-laden macrophages (Fig. 4). This finding was consistent with a diagnosis of PCH, a rare cause of pulmonary hypertension characterized by extensive proliferation of pulmonary capillaries within the alveolar septae. To confirm the presence of pulmonary hypertension, we then performed transthoracic echocardiography (TTE), however, there was no evidence of pulmonary hypertension, abnormality in cardiac wall motion, or valvular dysfunction. Thus, we concluded that the lung lesions were not PCH, but PCH-like foci.

**Figure 5:**
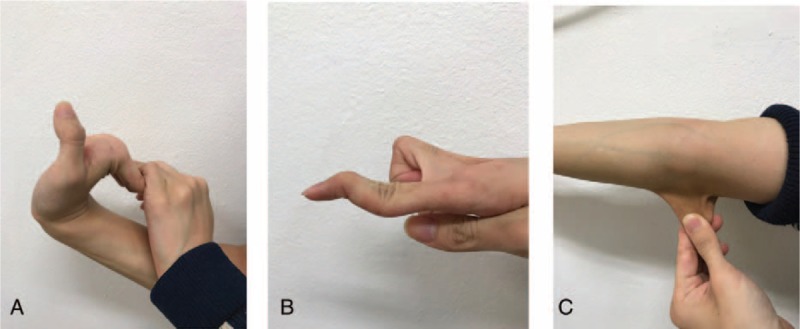
Skin and joint examination. Hyperflexibility of wrist and metacarpophalangeal joints (A) and swan-neck deformity of finger (B), and hyperextensible, thin, and transparent skin with vein visible (C) were suggestive of Ehlers–Danlos syndrome.

To find out underlying cause of all those atypical clinical manifestations, in-depth history taking and physical examination were done. The patient was tall and slender with height of 164 cm and weight of 45 kg (body mass index of 16.73 kg/m^2^). In addition, the joints were hypermobile especially in fingers and wrists, and the skin was hyperextensible and relatively transparent with veins that are visible (Fig. 5). Recurrent pneumothorax, abnormal elasticity of transparent skin, and hypermobility of small joints suggested a possible diagnosis of EDS. We therefore performed genetic test using his whole blood and c.1761 + 1G>A mutation causing skipping in exon 25 of *COL3A1* gene was identified. Finally, a definitive diagnosis of vEDS with concomitant PCH-like foci and cryptorchidism was made.

We then recommended genetic testing for his family members, and further imaging tests to evaluate possible arterial complications of central nervous system and abdomen. The patient's parents, however, declined to undergo the additional testing for economic reasons. He was discharged after removal of the chest tube on hospital day 45. The pulmonary function test performed before the discharge showed forced vital capacity of 2.83 L (78% of predictive value), forced expiratory volume in 1 second of 2.57 L (77% of predictive value), diffusing capacity of carbon monoxide of 57% of predictive value, and marginal bronchodilator response (10% and 230 mL change in forced expiratory volume in 1 second), which were compatible with CT-suggested emphysematous change and bronchial asthma. Mild eosinophilia shown in the initial laboratory finding could be explained by the latter. He did not need asthma medication as he showed no asthmatic symptoms or wheezing during the clinical course. As of 10 months after discharge, fortunately, the patient remains symptom-free and lives well, without relapse of pneumothorax and development of new pulmonary lesions or pulmonary hypertension.

## 6 Discussion

vEDS is autosomal-dominant and rare variant, accounting for approximately 4% of all EDS cases. The incidence of vEDS is estimated to be about 1 in 90,000.^[14]^ vEDS is caused by structural defects or deficiency of pro-alpha 1 chain of type III procollagen encoded by *COL3A1* which is an important component in many of the stretchable hollow organ tissues including arterial, bowel, the uterine walls, and skin. Thus, this abnormal type III collagen synthesis is associated with hyperextensibility of skin, joint hypermobility, and increased tissue fragility observed in patients with vEDS.^[1]^ In contrast to classic EDS which has features of generalized joint hypermobility and skin hyperextensibility, vEDS is characterized by thin transparent skin, easy bruising and hypermobility limited to the small joints.^[15]^ A clinical diagnosis of vEDS is usually based on 2 or more of the following criteria: characteristic facial features with a pinched nose, narrow lips, and prominent eyes; easy bruising and thin skin with visible veins; or tissue fragility with easy rupture of arteries and internal organs.^[3]^ A formal diagnosis of vascular EDS requires the demonstrated presence of a pathogenic mutation within the *COL3A1* gene.^[3,16,17]^

Numerous different types and sites of mutations in *COL3A1* have been identified to date. The correlation between clinical manifestation or prognosis and specific mutations was not established until recently. However, very recent studies showed evidence that the clinical features and prognosis are associated with specific mutation types.^[14,17]^ Frank et al,^[17]^ using 126 patients with molecularly proven vEDS, have reported phenotypic difference according to the variants of vEDS. They classified the population into 5 groups and found that digestive tract ruptures had occurred, exclusively, in glycine substitutions and splice-site mutation groups. In addition, patients with splice-site mutations were shown to be diagnosed at a significantly earlier age compared to those with glycine substitutions (25 vs 34 years). Interestingly, the lowest event-free survival, only 10% at the age of 40, was observed in splice-site variants.^[17]^ In terms of survival, Pepin et al^[18]^ analyzed a large US cohort which included 1231 individuals with documented diagnoses of vEDS and demonstrated that the median survival of vEDS was 51 years and was significantly less in males; the mortality rate is 2 times higher in males before age 20, due to frequent vascular rupture. Moreover, the patients with splicing donor site mutation showed a poorer prognosis (median survival of 37 years) than any other variants.^[18]^

The mutation detected in the present study, c.1761 + 1G>A in intron 24, is an extremely rare mutation which has been reported in only 1 previous case,^[17]^ and is correspondent to splice-site mutation. Although the genetic testing for his family members was not be performed, the mutation did not seem hereditary because his older brother, mother, and father (ages 25, 50, and 52-years-old, respectively) all denied any significant symptoms, or medical or surgical history. In fact, about 50% of *COL3A1* mutation is not inherited from a parent.^[19]^

The early evolution of the clinical events which ultimately leads to an early diagnosis (before the age of 20) is in line with a study mentioned above,^[17]^ while the potentially fatal arterial complications had not yet occurred. Moreover, investigators of a similar case of vEDS caused by splice mutations (c.1761 + 1G>A versus our case: c.1762 – 2A>G) in the *COL3A1* gene reported a rapidly progressing arterial aneurysm and early death of the patient due to a ruptured subclavian artery.^[20]^ Thus, it is important for his family to be informed about the future risk of vascular or surgical complications due to fragile tissues, the necessity of close follow-up for the potentially fatal complications of the condition and be strongly encouraged to receive genetic counseling.

The pulmonary complications of vEDS were reported to occur in 16% of vEDS, and they are generally not related to patient mortality.^[21]^ However, fatal cases of massive hemoptysis or diffuse alveolar hemorrhage have been reported.^[11,21]^ Representative cases which described pulmonary complications of vEDS are summarized in Table 1. In our case, the patient presented with recurrent spontaneous pneumothorax like several previous reports,^[5,8,18]^ but he also had lung parenchymal lesions-waxing and waning GGOs and newly developed cystic lesions predominantly in lower lobes and periphery which were diagnosed with PCH-like foci. Although bleeding complications due to pulmonary hemorrhage or intrapulmonary hematomas^[10,11,19]^ and pulmonary cysts and nodules^[8,17,22,23]^ were reported in previous reports, PCH-like foci has not been reported even in other variants of EDS. PCH is one of the rare causes of pulmonary hypertension. Histologically, it is characterized by extensive proliferation of pulmonary capillaries which is defined as at least 2 layers of aberrant capillaries within an alveolar septum and with evidence of endothelial markers, including CD31 or CD34.^[24]^ The extensive proliferation of pulmonary capillaries causes occlusion of the pulmonary vasculature, finally culminating in pulmonary hypertension.^[24]^ Radiologically, typical findings include diffuse bilateral diffuse centrilobular nodules with thickening of interlobular septa on HRCT.^[23]^ The common symptoms of PCH are progressive dyspnea and chest tightness which are nonspecific and can be seen in all forms of pulmonary hypertension.^[25]^ Its prognosis is very poor as evidenced by ineffective treatments for pulmonary hypertension.^[24]^ Although histopathological findings were compatible with PCH, the patient lacked the aforementioned clinical, cardiologic features. Consequently, PCH-like foci rather than PCH was diagnosed.

**Table 1:**
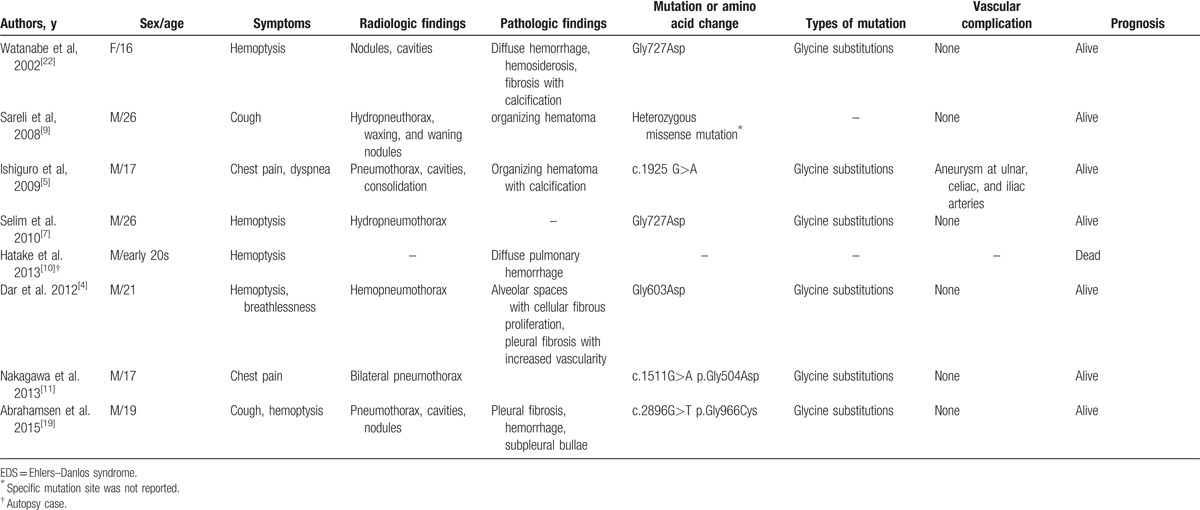
Summary of cases presented mainly with pulmonary complications in vascular EDS.

PCH-like foci has been rarely reported.^[26–28]^ Kadowaki et al^[28]^ reported a 55-year-old man who presented with multiple small GGOs discovered by chest CT and was then diagnosed with PCH-like foci. The histopathologic and radiologic findings – proliferation of capillaries in the alveolar septae coincided with multiple small GGOs in both lungs detected on HRCT – are similar to those of our case, although the patient was older than our case and the clinical course after the pathologic diagnosis was not described. Yost et al^[13]^ reported a case of fatal pulmonary hemorrhage which occurred in vEDS. The autopsy findings revealed diffuse alveolar hemorrhage and capillaritis and hemosiderosis. In other case by Dar et al, they reported a vEDS case presented with hemoptysis and hemopneumothorax, and described increased fibrosis on the pleural surface with an increased vascularity on surgical lung biopsy.^[15]^ Although the histopathologic findings of those 2 cases are partly similar to the findings revealed in our study, they were not associated with the abnormal capillary proliferation featured in the present case. It is not clear how this kind of lesion was developed in our case. We could speculate that abnormal proliferation of capillary endothelial cells and pleural fibrosis might be provoked by spontaneous and subtle pulmonary hemorrhages which appeared as multiple GGOs on chest CT scan, and which might be accelerated by mechanical trauma related with previous chest tube insertion, pleurodesis, or lung surgery.

Another atypical presentation, cryptorchidism, also has not been reported as a complication of vEDS. Instead, this phenomenon has been reported in a rare variant of EDS. ^[29]^ As we genetically tested all the possible genes and mutations related with several connective tissue diseases, we believe that we could rule out other genetic abnormalities or other variants of EDS. How the atypical clinical presentations could develop, and whether those phenomenons are truly associated with vEDS or incidental findings, requires further investigation.

This report has several limitations. First, the information on the arterial status of our case is not available as he refused further vascular work-up that we deemed critical for an accurate prognosis. Second, it is not clear whether he had a sporadic or inherited mutation, as we did not perform genetic testing on his family members. However, the strength of this report lies in the fact that we performed whole exome sequencing for his diagnosis; thus, we could rule out the possibility of coexistent genetic diseases or other variants of EDS.

In summary, we describe a case of vEDS with PCH-like foci and cryptorchidism which have not previously been described as complications of vEDS. His mutation belongs to splice-site variants and he was thus diagnosed at a relatively early age in accordance with a previous report.^[17]^ Although it is not evident whether he has any arterial complications, we can anticipate that he will remain very vulnerable to developing lethal vascular or vital organ complications compared to other variants of vEDS.^[18]^ This case emphasizes the importance of clinical suspicion for connective tissue disorders when we encounter atypical pulmonary manifestations including waxing and waning GGOs, atypical cysts and recurrent pneumothorax occurred especially in young adults. Also, individualized genetic counseling and clinical follow-up should be recommended to proactively address the future risk of complications, based on the recent evidence on genotype-phenotype association in this condition.
